# Yeast metagenomics: analytical challenges in the analysis of the eukaryotic microbiome

**DOI:** 10.20517/mrr.2023.27

**Published:** 2023-10-23

**Authors:** Sonia Renzi, Stefano Nenciarini, Giovanni Bacci, Duccio Cavalieri

**Affiliations:** Department of Biology, University of Florence, Sesto Fiorentino 50019, Italy.; ^#^Authors contributed equally.

**Keywords:** Yeasts, fungi, microbiome, microbial eukaryotes, eukaryome, ngs, metagenomics, taxonomy

## Abstract

Even if their impact is often underestimated, yeasts and yeast-like fungi represent the most prevalent eukaryotic members of microbial communities on Earth. They play numerous roles in natural ecosystems and in association with their hosts. They are involved in the food industry and pharmaceutical production, but they can also cause diseases in other organisms, making the understanding of their biology mandatory. The ongoing loss of biodiversity due to overexploitation of environmental resources is a growing concern in many countries. Therefore, it becomes crucial to understand the ecology and evolutionary history of these organisms to systematically classify them. To achieve this, it is essential that our knowledge of the mycobiota reaches a level similar to that of the bacterial communities. To overcome the existing challenges in the study of fungal communities, the first step should be the establishment of standardized techniques for the correct identification of species, even from complex matrices, both in wet lab practices and in bioinformatic tools.

## INTRODUCTION

In natural microbial systems, including host-associated microbiomes, microbial eukaryotes coexist with bacteria, archaea, and viruses, acting as decomposers, predators, parasites, and producers^[1]^. Theoretically, any ecosystem on Earth hosts eukaryotic microorganisms, from extremophiles in geothermal vents to endophytic fungi in plants to parasites or commensals with the gastrointestinal tracts of animals. In host- associated microbiomes, microbial eukaryotes implement complex interactions with their hosts: in plants, they defend the host against herbivorous organisms and enhance nutrients assimilation^[2]^; in animals, they can metabolize plant compounds in the host’s gastrointestinal systems^[3]^. However, both plants and animals can also be afflicted by microbial eukaryotes^[4,5]^. In humans, microbial eukaryotes interact with the host immune system in intricate ways. The low diversity in microbiomes from industrialized countries reflects the “extinction” reported for bacterial communities, which is a result of globalization^[6-8]^. Beyond host interactions, microbial eukaryotes are essential to the ecology of aquatic and soil ecosystems, where they serve as primary producers, symbiotic partners, decomposers, and predators^[9,10]^.

Fungi constitute the group of eukaryotes with the highest diversity and global distribution. Thanks to a wide range of morphological, physiological, and ecological features, these organisms have evolved to colonize the most diverse ecosystems^[11]^. Within the fungal kingdom, yeasts are not strictly identified, as the term refers to a unicellular lifestyle that has evolved several times rather than a taxonomic unit^[12]^. Yeasts and yeast-like fungi are the most prevalent eukaryotic components of the microbiota due to their ubiquity, yet their abundance and influence are frequently underestimated.

Despite their relevance, eukaryotic microorganisms are generally largely neglected in microbiome investigations^[13]^. Traditionally, culture-based techniques have been employed to explore and study microbial diversity and to obtain a representative set of isolates based on physicochemical variation. However, due to intrinsic methodological limitations, this approach has been progressively replaced by culture-independent ones, although it has been rediscovered and subjected to various refinements in recent years to enable the capture of a broader spectrum of microorganisms^[14,15]^. Following the advent of Sanger sequencing, the use of DNA for the identification of microorganisms has become standard practice, revolutionizing microbial genotyping and taxonomy^[16,17]^. The most recent rise of second- and third-generation sequencing approaches has facilitated the advancement of eukaryotic-specific amplicon sequencing, which is revolutionizing our understanding of the eukaryotic diversity in host-associated and environmental microbiomes^[18-22]^. Like all amplicon-based techniques, this approach can suffer from poor taxonomic precision and difficulty discriminating between closely related species^[23,24]^. In contrast, whole metagenome sequencing captures DNA from the entire pool of species present in a microbiome, including eukaryotes, without the need for experimental selection. Whole metagenome sequencing data are becoming predominant in microbiome research because they can be used to assemble unknown genomes, classify strains, and assess the presence or absence of genes and pathways^[25]^. These methods are useful for identifying bacteria and archaea, but microbiome-associated eukaryotes, such as yeasts, are still difficult to detect, especially in large metagenome sequencing datasets. One of the main reasons for this issue is that despite being part of one of the largest branches of the “Tree of Life”, the number of high-quality fungal target sequences or genomes in curated databases is still significantly lower than that of available bacterial ones, severely limiting the possibility of properly investigating these organisms.

The aim of this review is to outline the current state of research regarding the techniques and experimental pipelines for the study of yeast metagenomics, focusing on the currently unresolved methodological challenges as well as the pros and cons of each different approach.

## MYCOBIOME: FOCUS ON YEASTS

The term “mycobiome”, coined in 2009^[26]^ for a study of fungal communities on salt marsh plants using molecular fingerprinting, was then used in 2010 to refer to the human oral mycobiome^[27]^, Now, it is used to indicate the fungal component of every microbial ecosystem. Within the fungal kingdom, the term “yeast” is used to describe any fungus that reproduces asexually by budding or fission, produces single-cell stages, and has sexual structures that are not enclosed in a fruiting body^[28]^. This broad description is frequently used to encompass dimorphic lineages that produce mycelial growth in their sexual phases, as well as biotrophic diseases and black yeasts. As a result, they do not constitute a taxonomic unit but rather a lifestyle shared by multiple distinct lineages, even though there are several exceptions and comments to the labile border between yeasts and dimorphic filamentous fungi that produce yeast-like stages, along with yeast lineages that grow solely as filamentous, are outlined^[29]^.

Yeasts occur in the division Ascomycota, mainly in the subdivisions Saccharomycotina (so-called budding yeasts) and Taphrinomycotina (that also includes so-called fission yeasts), as well as in three subdivisions of Basidiomycota, namely Ustilaginomycotina, Pucciniomycotina, and Agaricomycotina^[30]^.

These unicellular organisms have become popular in a various applications, including baking, brewing, winemaking, distilling, and an assortment of other conventional and non-conventional fermentations. They also serve as a versatile tool in biotechnology^[31]^, encompassing some of the most widely used model species (e.g., *Saccharomyces cerevisiae*, *Schizosaccharomyces pombe*, and *Candida albicans*). The rapid expansion of scientific understanding of yeast diversity is attributed to the uncovering of new species in nature and the use of specific identification tools like nutritional tests, biochemical and molecular characterizations, and DNA barcode technology. As a result of this technological advancement, previously identified fungal species are continuously reevaluated, and the concept of yeast species itself is evolving^[32]^.

According to existing estimates, only a small fraction (about 5%-10%, depending on the environment) of the entire variety of fungi has been identified^[33,34]^. It is estimated that Earth hosts between 2.2 and 3.8 million fungal species^[35]^, yet only about 4% of these are cataloged^[36]^. This situation likely holds true for yeast as well. Out of the approximately 150,000 fungal species described so far^[37]^, only around 2,000 are yeasts. The mycobiome is often neglected, both due to its lower abundance compared to bacteria and the methodological challenges associated with its detection^[38]^.

The high incidence of cryptic and hybrid species hampers efforts to accurately quantify species diversity. These issues have long been acknowledged, but the advent of whole-genome sequencing has brought them to the forefront^[39]^. In fact, when speaking about genomes, fungi exhibit more complex genetic features compared to bacteria, including multiple chromosomes, expanded repeated regions, and larger genome sizes, all of which introduce inaccuracies during sequence classification. Therefore, there is a need for comprehensive benchmarking of both classification algorithms and databases to optimize identification pipelines for the fungal kingdom.

## CHARACTERISING THE MYCOBIOME: IDENTIFICATION AND TECHNOLOGICAL ISSUES

As mentioned above, many questions regarding mycobiota remain to be addressed. Several methodologies commonly applied for the investigation of the bacteriome are not consistent when used for studying the fungal community. Consequently, non-standardized techniques, technical challenges, restricted availability of reference data, and other issues have emerged^[40]^. Therefore, it is crucial to enhance our knowledge and expand the spectrum of available technologies in order to address the challenges posed by the fungal communities inhabiting the environmental ecosystem and our bodies.

### Culture-dependent approaches

Traditionally, culture-dependent approaches been employed to investigate microorganisms’ diversity, including fungi. However, these techniques have well-known limitations. For instance, many species remain undetected because appropriate culture conditions are either unknown or challenging to reproduce^[41]^. Moreover, culture methods are time-consuming and hardly suitable for high-throughput analysis. Culturomic approaches offer undeniable benefit as they provide access to the fungus itself, allowing for the assessment of its viability, metabolites, phenotypical and functional characterization, and other host-adaptation features^[42]^. In recent years, the integration between culture- dependent and culture-independent approaches has increased, thanks to molecular techniques. Sequencing of large portions or entire microbial genomes has provided the necessary information for fine-tuning the growth conditions of even those microorganisms considered “unculturable” until a few years ago^[43,44]^. As a result, culture-dependent approaches remain useful and of great interest^[45,46]^. This is especially important given that some fungal strains cannot be accurately identified by a culture-independent method. This underrepresentation of some species might result from factors such as cell wall structure or the inadequacy of the chosen PCR primers and/or barcode sequence^[47]^. However, the identification process for isolated fungal strains is not yet complete and requires further steps, often involving culture-independent approaches.

### Culture-independent approaches

**Figure 1:**
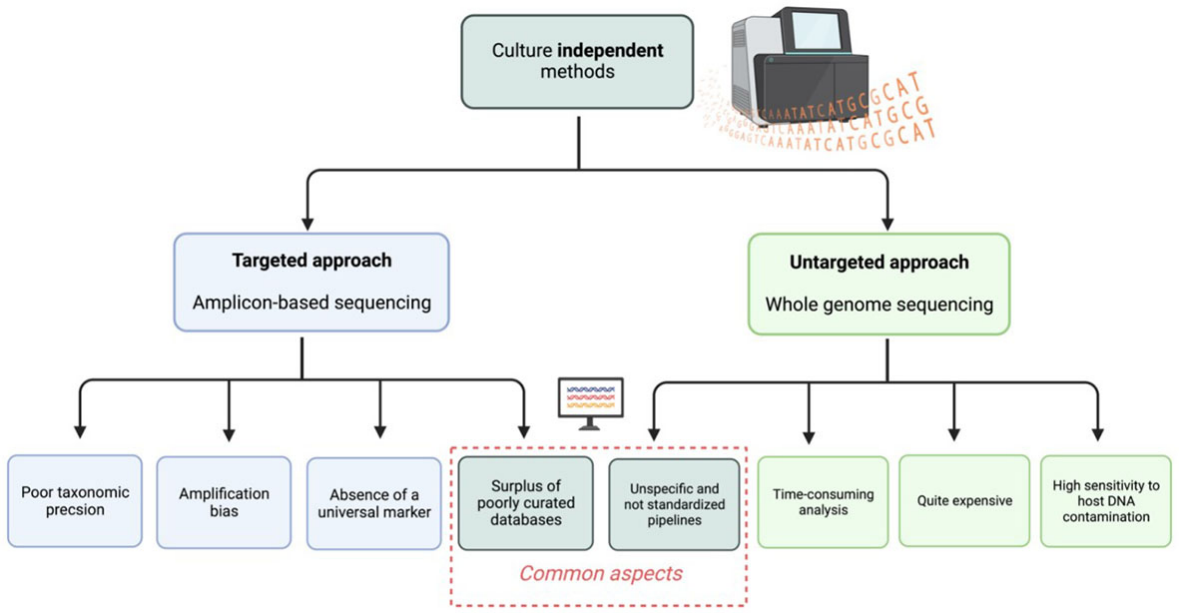
Schematic representation of current limitations in culture-independent methods.

The use of DNA as an identifying marker in culture-independent approaches avoids some of the aforementioned issues. However, this method strongly relies on the choice and efficiency of DNA recovery methods, and it also introduces new limits and hurdles [Figure 1]. Fungi, unlike bacteria, have a strong and complex cell wall rich in glucans and chitin^[48-51]^. Consequently, the efficient destruction of the fungal cell wall is crucial for genomic DNA extraction. Several bead-beating stages followed by enzymatic cell lysis are required for successful mycobiota analysis of any sample matrix^[47]^. Following DNA extraction, different approaches can be used to detect and identify fungi. This methods may include PCR^[52]^, metabarcoding sequencing analysis, or whole genome sequencing (WGS) metagenomics.

#### Amplicon-based sequencing: a matter of target

While amplicon sequencing techniques have successfully revealed the microbiome of a plethora of organisms^[53-56]^, the choice of the marker to use is crucial as it drastically affects the type of organisms that can be detected. In the micro-eukaryotic world, mainly composed of fungi, protists, algae, and other microorganisms known to inhabit almost all ecological niches explored on Earth, the selection of “universal” targets is limited [Table 1]. Only a few available pipelines are available to cope with markers different from the well-known bacterial 16S rRNA gene^[57-60]^.

**Table 1:** List of barcode loci for fungal taxonomic identification

**Genomic locus**	**Proposed by**	**Ref.**
ITS1-4 (whole region)	Schoch *et al.*	[24]
ITS1 as preferred for Basidiomycota identification	Bellemain *et al.*	[68]
ITS2 as preferred for Ascomycota identification	Bellemain *et al.*	[68]
ITS2 as preferred for human mycobiota identification	Hoggard *et al.*	[70]
ITS2 subregion	Nilsson *et al.*	[62]
TEF1α	James *et al.*	[77]
TOP1	Stielow *et al.*	[76]
PGK	Stielow *et al.*	[76]
RPB1	Matheny *et al.*	[78]
RPB2 for environmental fungal communities	Větrovský *et al.*	[80]
IGS	Morrison *et al.*	[81]
β-tubulin	Geiser *et al.*	[82]
LSU (D1/D2 region)	Kurtzman and Robnett	[72]

IGS: Intergenic spacer; LSU: large subunit; PGK: phosphoglycerate kinase; TOP1: topoisomerase I.

Similarly to bacterial metabarcoding, the usual fungal barcode is the rRNA gene locus, which includes the genes for 18S rRNA, 5.8S rRNA, and 28S rRNA, separated by the internal transcribed spacers (ITS1 and ITS2). This approach seems to discriminate better at higher taxonomic ranks than the 16S rRNA gene^[61]^. After exploring fungal rRNA genes, Schoch *et al.* in 2012 identified the ITS as the possible universal DNA barcode identifier for fungi^[24,62]^, although currently, it is still not clear which of the two ITS components has the better resolution in strain prediction. Recent findings has shown that both regions suffer from amplification biases, resulting in an uneven representation of synthetic fungal communities^[63-65]^: ITS1-based PCR appears to favor Basidiomycota, whereas Ascomycota seems to be favored by ITS2-based PCR^[66-68]^, although this consideration should not be generalized. In fact, there are known ascomycetes species (such as the ones belonging to the genera *Saccharomyces* and *Komagataella*) that are discriminated with greater resolution by employing the ITS1 marker^[69]^. Hoggard *et al.* recommend the selection of the ITS2 region in human mycobiota investigation after comparing four sets of primers targeting the small subunit (SSU) rRNA (18S), ITS1, ITS2, and large subunit (LSU) rRNA (26S) genomic regions^[70]^. In yeast, the D1/D2 region of the LSU gene cluster within the ribosomal DNA (rDNA) has been a longstanding and effective tool for species identification and strain differentiation, pre-dating the conceptualization of DNA barcoding^[71,72]^. In addition, Nilsson *et al.* propose a set of fungus-specific primers with superior coverage of the fungal kingdom, targeting the ITS2 sub-region with degenerate forward primers gITS7ngs and a reverse primer ITS4ng^[61]^. Besides primers’ choice, length variation among ITS sequences from fungal species, spanning from 200 to 800 bp, has a strong impact on PCR efficiency as well as sequencing technologies^[73,74]^. Moreover, not only is this region present in multiple copies within one species^[75]^, but intragenomic variation within a single species, such as numerous paralogous or non-orthologous copies, may lead to an overestimation of global fungal diversity^[24]^. Since the ITS copy number has highly interspecific variation, an accurate determination of fungal abundance is hard to reach, and quantitative comparisons between diverse species in mixed populations must be made with caution. The lack of universal taxonomic resolution and the potential presence of non-homologous ITS copies in the genome made the identification of supplementary molecular markers necessary. Using *in silico* pipelines, Stielow *et al.*^[76]^ confirmed the already known TEF1α^[77]^ as a secondary barcoding marker for fungi and proposed the genes topoisomerase I (TOP1) and phosphoglycerate kinase (PGK) as promising ascomycetes identifiers based on the analysis of complete sequenced genomes^[76]^. Other suggested secondary markers for fungal DNA amplification are the intergenic spacer (IGS), RNA polymerase II (RPB1 and RPB2), β-tubulin II (TUB2), and the minichromosome maintenance complex component 7 (MCM7) protein^[78-82]^. The selection of one or more reference genes is crucial for standardization and promotion of large-scale investigations, but in some cases, primer bias in targeted sequencing can be overcome by opting for the shotgun metagenomic approach.

#### Metagenomic whole genome sequencing

Shotgun metagenomic sequencing allows for a higher taxonomic resolution as it sequences most of the genomes of every organism present within a sample^[83]^. This capability not only to identifies the organism but also characterizes extended profiles, including antimicrobial resistance, genetic subtypes, metabolism, and virulence^[84]^. Despite being a highly effective method for describing pathways and discovering novel functions, shotgun metagenomics is significantly more expensive and computationally more intensive than amplicon sequencing, depending on sequencing depth^[85]^.

Moreover, due to its non-specificity, WGS is the most unbiased technique but also the most sensitive to host DNA contamination, especially in soft tissues and biological fluid samples where host DNA can dominate the sequenced reads^[86]^. This sensitivity is a significant concern for the study of mycobiota since fungi represent only a small fraction of the total microbial biomass. Achieving adequate sequencing depth is required to perform the analysis. Currently, it appears that low fungal abundance in human samples is impeding the broad use of metagenomic WGS in human samples, a finding that is unrelated to DNA extraction techniques and reflects really low total in vivo fungal abundance^[87]^.

The development of high-throughput sequencing techniques has greatly benefited our understanding of microbial ecology. Nevertheless, the most common methods currently in use, which produce short reads, often suffer from limited species-level resolution and identification uncertainty. Fortunately, recent developments in long-read sequencing technologies by PacBio and Oxford Nanopore are enabling the reconstruction of more complete fungal genomes. These long reads, often exceeding 10 kb in length, can cover critical genomic regions, including highly repetitive ones^[84,88-91]^.

Using long-read sequencers, researchers have successfully generated whole genomes of major pathogenic fungi, often in combination with short-read sequencing, a technique known as hybrid assemblies^[92-99]^.

### Bioinformatics

In metagenomics and metabarcoding analyses, data interpretation is a significant challenge. While these approaches enhance the objectivity of fungal phylogeny and subsequent accurate identification, they simultaneously generate ever-growing amounts of sequencing data. Addressing the prompt delivery of the enormous amount of sequence data available to end user introduces a new challenge.

#### Databases: need for unification

Thanks to advancements in computational technology and bioinformatics tools, large volumes of data can now be easily stored, annotated, and accessed remotely with relative ease. As a result, a surplus of nucleotide sequence databases for fungal studies was created^[23]^. The strategic value of a database is based on its accessibility, through which end users may deposit, save, annotate, and retrieve data. It must be considered that every database has an intrinsic proclivity to become outdated over time. To maintain useful and relevant databases for diagnostics and research, a dedicated group of trained professionals is required to carry out an ongoing and systematic curation. Over the last decade, many online fungal databases have been established for the mycology research community. However, not all of them have a dedicated team of curators or an updated maintenance system. Some of the most widely used repositories [Table 2], such as Aspergillus Genome Database (AspGD)^[100]^, Barcode of Life Data Systems (BOLD)^[101]^, Broad Institute databases (http://www.broadinstitute.org/scientific-community/data/), Candida Genome Database (CGD)^[102]^, Comprehensive Yeast Genome Database (CYGD)^[103]^, Ensembl Fungi (https://fungi.ensembl.org), FungiDB^[104]^, FUNGIpath^[105]^, Fusarium-ID^[106]^, Fusarium Multilocus Sequence Typing (MLST)^[107]^, International Society for Human and Animal Mycology-Internal Transcribed Spacer (ISHAM-ITS)^[108]^, International Society for Human and Animal Mycology - MultiLocus Sequence Typing (ISHAM-MLST) (http://mlst.mycologylab.org/), JGI MycoCosm^[109]^, NCBI GenBank (https://www.ncbi.nlm.nih.gov/genbank/), NCBI RefSeq (http://www.ncbi.nlm.nih.gov/refseq/), PomBase^[110]^, Saccharomyces Genome Database (SGD)^[111]^, and UNITE^[112]^ have been resumed and extensively classified by Prakash *et al.*^[113]^. To avoid the hampering issues of comprehensive data management, they suggest a cloud-based, dynamic network platform based on the integration of particular focused-group databases with maximum access and functional characteristics for the user community.

**Table 2:** Principal genomic databases described according to their ability to discriminate fungal sequences

**Database**	**Ref.**	**Description**	**Taxonomic discriminative potential**
AspGD	[100]	AspGD focuses on the genomes of Aspergillus species. It provides detailed genomic data, including gene annotations, functional information, and comparative genomics. Enables the identification of both the species and strain levels.	High within the genus Aspergillus
BOLD	[101]	BOLD is a comprehensive online platform primarily dedicated to DNA barcoding and biodiversity research. While it is a valuable resource, its primary focus is on animal barcoding. As a result, its fungal taxonomic discriminative potential is limited compared to databases specifically tailored for fungi.	Limited
Broad Insitute Database	http://www.broadinstitute.org/scientific-community/data/	The Broad Institute has contributed extensively to fungal genomics. It offers genomic data for a variety of fungal species, with an emphasis on pathogenic fungi.	High
CGD	[102]	CGD is dedicated to *Candida* species, and it offers genomic sequences, gene annotations, and pathogenicity-related information, supporting research on the genus *Candida*.	High within the genus Candida
CYGD	[103]	CYGD offers comprehensive genome annotation and functional data primarily for *Saccharomyces cerevisiae*. While it provides essential information for yeast research, its taxonomic scope is restricted to this species.	Limited to species *S. cerevisiae*
Ensembl Fungi	https://fungi.ensembl.org	Ensembl Fungi is a component of the Ensembl project, offering genomic data and tools for various fungal species. While it covers a range of fungi, it may be more comprehensive for some taxa than others.	Moderate
FungiDB	[104]	FungiDB is a genomic database focused on fungal pathogens. It includes a diverse set of fungal genomes, with an emphasis on medically important species.	Moderate
FUNGIpath	[105]	FUNGIpath is a resource for fungal pathogen genomics. It provides genomic sequences and annotations for pathogenic fungi, with relevance to disease research.	Moderate
Fusarium-ID	[106]	Fusarium-ID is a specialized database for *Fusarium* species identification and classification. It provides detailed molecular and phenotypic data for various Fusarium species, including pathogenic strains.	High within the genus *Fusarium*
Fusarium MLST	[107]	Fusarium MLST is a database that focuses on sequence-based typing for *Fusarium* species. It allows researchers to differentiate between closely related *Fusarium* isolates by analyzing multiple gene loci. This database is particularly useful for studying genetic diversity within the genus.	High within the genus *Fusarium*
ISHAM-ITS	[108]	ISHAM-ITS database is designed to aid in the identification and classification of medically important fungi using the fungal Internal Transcribed Spacer (ITS) region of ribosomal DNA. Its taxonomic discriminative potential is high within the context of identifying and characterizing fungi relevant to human and animal health.	High within the medical mycology
ISHAM-MLST	http://mlst.mycologylab.org/	ISHAM-MLST is dedicated to the Multilocus Sequence Typing of medically important fungi, particularly those associated with human and animal mycoses. It has a higher taxonomic discriminative potential for distinguishing between closely related strains within a species.	Very high within the medical mycology
JGI MycoCosm	[109]	MycoCosm, hosted by the JGI, provides access to a diverse collection of fungal genomes, including those from various taxonomic groups, making it suitable for discriminative research.	High
NCBI GenBank	https://www.ncbi.nlm.nih.gov/genbank/	NCBI GenBank is a comprehensive and widely used repository for genomic data. It covers a wide taxonomic range, including fungi, but the level of detail and annotation quality can vary.	Moderate to high
NCBI RefSeq	http://www.ncbi.nlm.nih.gov/refseq/	NCBI RefSeq offers high-quality genomic annotations and reference sequences, making it a preferred choice for researchers seeking accurate taxonomic and functional information for well-studied fungal species.	High
PomBase	[110]	PomBase is primarily focused on *Schizosaccharomyces pombe*. It provides detailed genomic and functional information for this species, making it an excellent resource for S. pombe research. However, its taxonomic scope is limited to this species.	Limited to species *S. pombe*
SGD	[111]	SGD is dedicated to *Saccharomyces cerevisiae* and is a comprehensive resource. While its primary focus is *S. cerevisiae*, it contains extensive genomic and functional data that can support the study of other Saccharomyces species as well.	High within the genus *Saccharomyces*
UNITE	[112]	UNITE provides a comprehensive collection of fungal ITS sequences, covering a broad range of fungal taxa, from common and well-studied species to rare and less-known fungi.	High

AspGD: Aspergillus genome database; BOLD: barcode of life data systems; CGD: candida genome database; CYGD: comprehensive yeast genome database; ISHAM-ITS: international society for human and animal mycology-internal transcribed spacer; ISHAM-MLST: international society for human and animal mycology - multilocus sequence typing; JGI: joint genome institute; MLST: multilocus sequence typing; SGD: saccharomyces genome database.

One of the most concerning analytic challenges in mycobiota investigations is the inadequate curation of fungal databases. This deficiency in high-quality fungal sequences within curated databases results in a substantial number of unclassified reads. Addressing this issue may involve producing additional high-quality metagenomic and whole-fungal genome assemblies^[87]^. Furthermore, sequencing data are frequently devoid of any biologically relevant information, such as the substrate of origin or details on the technology used. Thus, well-curated fungal databases with accurate sequence data play a pivotal role in further research and diagnostics in the field of mycology. The current fungal databases only poorly represent the diversity of the fungal kingdom, limiting their analytical power.

#### Pipelines

The bioinformatics analysis workflow for amplicon data can be summarized into four main steps: (i) pre- processing; (ii) “grouping” of amplicon sequences; (iii) taxonomic classification; and (iv) visualization and statistical analysis^[114]^. While various tools can be used in each of these steps, producing slightly different results, the second step, in particular, is crucial. Amplicon sequences can be clustered based on their similarity^[115-119]^, akin to classical clustering techniques such as k-mean clustering or agglomerative clustering - or based on single nucleotide differences across them, an approach currently known as sequence variant inference^[60]^. Methods falling into the first category profile bacterial communities by grouping similar sequences into Operational Taxonomic Units (OTUs), but the definition of a similarity threshold has always been empirical. As a consequence, these methods tend to produce a large number of OTUs that are not always biologically relevant, an issue that goes by the name of “OTU inflation”^[120]^. This massive production of OTUs may lead to wrong conclusions and/or to the generation of huge datasets, which can be difficult to analyze. Tackling this issue is not trivial, and a series of novel approaches have been proposed. These approaches rely on the definition of sequence variants from single nucleotide differences in the amplicon reconstruction, trying to profile microbial communities based on “real” differences instead of sequence similarity. Nowadays, the research communities are gradually moving to the new concept of Amplicons Sequence Variants (ASVs) or Exact Sequences Variants (ESVs)^[121]^ for profiling bacterial communities, and it should also be recommended for yeasts and yeast-like organisms. These approaches generate an error model for each sequencing run, which enables discriminating between a true sequence variant (i.e., one sequence with a single SNP with respect to another) from sequencing errors^[60]^. Since these processes rely on the single nucleotide variation of amplicons for defining taxonomy, they usually lead to an increased estimation of alpha diversity, mainly due to their higher sensitivity with respect to identity-based approaches. One of the greatest assumptions of these methods is that the amplicon sequence should not vary in length, and ITS sequences from fungi do not share this assumption. This may lead to biases in the discriminatory potential of these methods, even if, at present, no extensive survey has been performed^[122]^. To reduce these biases, a number of ITS sequencing-based systems have been created to identify different fungal species. Some of them are able to examine both 16S rRNA (from bacteria) and ITS (from fungi), such as Kraken^[123]^, Mothur^[115]^, Qiime^[119,124]^, Vsearch^[117]^, and DADA2^[60]^; others are specialized on fungi only, such as Plutof^[125]^, Clotu^[126]^, PIPITS^[116]^, CloVR-ITS^[127]^, MICCA^[128]^, and BioMaS^[129]^. Despite these well-known issues, standardized pipelines are still to come, leaving the choice of the analysis method in the hands of researchers. This situation opens a whole new scenario where researchers are responsible for the pipeline they used (which, in most cases, is published and freely available), and this choice may alter the research outcomes^[130]^, paving the way for contrasting conclusions. Although pipelines based on the bacterial 16S gene (or part of it) have been extensively used in the last three decades, the “yeast world” remains largely unexplored, and the effect of one pipeline compared to another is unpredictable. A summary of the main pipelines available is reported in Table 3^[60,115-117,119,123,124,126-129,131,132]^.

**Table 3:** List of currently available pipelines for meta-barcoding

**Name**	**Clustering algorithm**	**Yeast-specific**	**Ref.**
Clotu	Identity-based clustering	Yes	[126]
PIPITS	Identity-based clustering	Yes	[116]
CloVR-ITS	Identity-based clustering	Yes	[127]
BioMaS	Reference-based	No	[129]
Kraken	Reference-based	No	[123]
Mothur	Mixed	No	[115]
Qiime (1 & 2)	Mixed	No	[119,124]
MICCA	Mixed	No	[128]
Vsearch	Identity-based	No	[117]
Uparse	Identity-based	No	[131]
Unoise (1 & 2)	Variant-based	No	[132]
DADA2	Variant-based	No	[60]

Clustering algorithms were divided into: (1) Identity-based, those relying on an empirical percentage of identity between two sequences for grouping them into a single cluster; (2) Reference-based, algorithms which group sequences into taxonomic bins according to their identities; (3) Variant-base, those defining sequence variants according to the presence of SNPs or mutations; (4) Mixed, pipelines which contain different algorithms for clustering.

In the context of metagenomic WGS, two primary strategies are commonly employed to analyze raw data: the alignment-based approach and the assembly-based approach. The first one involves mapping individual sequencing reads to a reference database or a reference genome. On the other hand, the second approach assembles reads de novo to form contigs, which are then clustered into so-called genome bins during a binning process. Combining both approaches is frequently advocated for result accuracy^[84]^. By now, many bioinformatic tools are available. Alignment-based tools are strong in taxonomic profiling and identifying known microorganisms. They include a step of fragment recruitment in order to map all the reads to one or more selected references. Among taxonomic profilers, MetaPlhAn2^[133]^, Kraken2^[134]^, and DIAMOND^[135]^ stand out for different skills. If you need high specificity and rapid analysis, MetaPhlAn2 might be a good choice. For comprehensive database coverage and strain-level resolution, Kraken 2 is valuable. DIAMOND allows customization and offers fast alignment capabilities, but it requires additional steps for taxonomic profiling. Assembly-based tools, instead, are essential for discovering novel organisms and in-depth functional analysis within metagenomic communities. Their workflow includes an assembler^[136]^ that is well suited for the reconstruction of long contigs and a genome binner to cluster such sequences from the same organism^[137]^. When selecting an assembler for WGS data, the type of sequencing technology used, the genome size, the desired level of assembly completeness, and the availability of computational resources should be taken into consideration. MetaSPAdes^[138]^, MegaHit^[139]^, and IDBA-UD^[140]^ are the most popular metagenome assemblers, also for fungal genomes. As well as for assemblers, there is no binning tool designed exclusively for fungal sequences, so general metagenomic binners are being used, like METABAT2^[141]^, CONCOCT^[142]^, MaxBin 2.0^[143]^ and MetaWrap^[144]^ to name a few of the most efficient. Many researchers also employ hybrid assembly strategies that combine short-read and long-read data to achieve more accurate and complete genome assemblies^[95]^. To delve deeper into the metagenomic data beyond taxonomic composition, functional annotation becomes necessary. Fragment recruitment, as previously described, involves leveraging a database of functionally annotated genes or proteins. This approach provides a straightforward means to achieve functional annotation. Subsequently, annotations showing a specific level of coverage can be linked to various aspects, such as metabolic pathways, with tools like KEGG^[145]^. Metagenomic WGS of fungi offers valuable insights into complex fungal communities, but it also comes with several drawbacks and challenges. Bioinformatic complexity, functional annotation, short-read sequencing, not standardized pipelines, data volume and processing are probably the main ones. Addressing these drawbacks often requires a combination of improved sequencing technologies, more comprehensive reference databases, advances in bioinformatics methods, and careful experimental design to mitigate potential biases and methodological limitations.

## CONCLUSION

In conclusion, fungi play a pivotal role in shaping diverse ecosystems, and while our understanding of their importance has grown considerably, there remain numerous avenues for exploration within the fungal kingdom. The advent of DNA-based classification methods has ushered in a transformative era in mycology, revolutionizing traditional taxonomic approaches while also providing robust validation of species identities. Despite significant progress, challenges persist in the field of fungal genomics. Sequencing techniques have revealed biases and limitations, particularly in fungal markers amplification. Recent innovations like long-range amplification and long-read sequencing hold promise for more accurate fungal classifications. The increasing availability of whole-genome shotgun sequencing and expanding genome databases offer opportunities to map newly generated fungal DNA sequences directly to comprehensive references.

Advancements in sequencing technologies are complemented by the development of taxonomic classification algorithms, but critical gaps remain. Benchmarking long-read sequencing strategies for fungal communities lags behind bacterial community studies. Similar disparities exist in the relative maturity of bioinformatic platforms and databases. Fungi’s unique complexities, such as multiple chromosomes, extended repeat regions, and larger genome sizes, add to the challenges.

The intricacies of fungal taxonomy further complicate identification efforts. The absence of standardized pipelines for sequencing data analysis remains a significant hurdle in mycobiota investigations. Given these challenges and opportunities, it’s evident that fungal research continues to rapidly evolve. Future progress will hinge on collaborative efforts to address existing gaps, harmonize methodologies, and advance our understanding of these essential and enigmatic organisms in the intricate network of global ecosystems.

## DECLARATIONS

### Copyright

© The Author(s) 2023.
